# Knowledge and information levels and adherence to oral anticoagulant therapy with warfarin in patients attending primary health care services

**DOI:** 10.1590/1677-5449.012017

**Published:** 2018

**Authors:** Thais Furtado de Souza, Christiane Fátima Colet, Isabela Heineck

**Affiliations:** 1 Universidade Federal do Rio Grande do Sul – UFRGS, Programa de Pós-graduação em Assistência Farmacêutica, Porto Alegre, RS, Brasil.; 2 Universidade Federal do Rio Grande do Sul – UFRGS, Programa de Pós-graduação em Ciências Farmacêuticas, Porto Alegre, RS, Brasil.

**Keywords:** warfarin, primary health care, information, medication adherence, international normalized ratio

## Abstract

**Background:**

Oral anticoagulation therapy with warfarin is widely used around the world and its safety and efficacy are well-established. Nevertheless, anticoagulants are among the drug classes most associated with fatal medication errors in primary health care.

**Objective:**

To investigate patient knowledge, the level of information provided, and medication adherence in patients treated with warfarin at a primary health care service.

**Method:**

A cross-sectional study of a prospective cohort of 60 patients on warfarin treatment in the town of Ijuí, Rio Grande do Sul, Brazil. A questionnaire was administered to test patients’ knowledge about their prescriptions and the level of information provided by the health team. The 8-item Morisky Medication Adherence Scale (MMAS-8) and International Normalized Ratio (INR) were used to verify adherence to treatment.

**Results:**

The results were expressed in absolute and relative values and prevalence ratios were calculated, with respective 95% confidence intervals. It was found that 83.3% of the participants had been given insufficient information by the health team, 50% did not know how to use the medication correctly, 86.7% were not adherent to the treatment according to MMAS-8 and 63.3% were outside of the correct INR range.

**Conclusion:**

In this study, we observed a need to improve the quality of information provided to users and to develop strategies to improve adherence to treatment, to ensure the safety of patients treated with warfarin in primary health care services.

## INTRODUCTION

 Warfarin oral anticoagulation is taken by millions of people worldwide and its safety and efficacy are well-established. 1 ^,^ 2 However, it demands rigorous clinical and laboratory monitoring and assessments by a multidisciplinary team. 3 Patients must regularly monitor blood coagulation levels with prothrombin time (PT) test results, which are expressed by the International Normalized Ratio (INR) 4 and must be kept within the established therapeutic range to reduce the risk of thromboembolic or hemorrhagic complications. 4

 In primary health care (PHC), oral anticoagulants (OACs), and warfarin in particular, are among the classes of medications most associated with fatal medication errors, which are very often caused by inadequate laboratory monitoring, significant drug interactions, gaps in the technical knowledge of the professionals involved, and insufficient patient guidance. 5

 Success and safety of OACs are both dependent on patient education, good adherence to treatment, and communication between patients and the teams responsible for their clinical care. 2 ^,^ 5 However, publications about OACs primarily emphasize adverse events, such as hemorrhages and thromboembolic events, without mentioning the quality of care. 5 In Brazil, studies of patient knowledge or adherence to oral anticoagulation treatment have focused on patients treated at specialist clinics. 3 ^,^ 4 ^,^ 6 ^-^ 8

 In this context, the present study was conducted with patients on oral anticoagulation treatment with warfarin who were not being seen at a specialist clinic, but were being treated by the PHC network in the town of Ijuí, RS, Brazil. The objectives were to determine users’ level of knowledge about warfarin prescriptions, the level of information about treatment precautions provided to these users by the healthcare team, and their adherence to treatment with warfarin. 

## METHODS

 A cross sectional study was conducted of a prospective cohort, with data collection between April and July of 2014. The patients in the cohort were seen monthly for a period of 18 months from April 2014 to October 2015. The sampling strategy was to recruit all patients who obtain their warfarin medication at health services in the municipal district of Ijuí. Ijuí has a population of 79,396 inhabitants 9 and the municipal PHC system has 15 units where medications dispensed: seven Basic Health Units and eight Family Health Strategy Units. 

 Patients taking warfarin were identified from prescriptions dispensed at the municipal district’s health units. This was accomplished by analyzing prescriptions that are filed at the municipal district’s Central Pharmacy to identify warfarin prescriptions. 

 Data collection was conducted using a questionnaire covering sociodemographic characteristics and also containing questions related to treatment with warfarin. Patients’ level of knowledge about their medical prescriptions was probed using an open question, asking interviewees to explain how they should use their medication. Interviewees’ answers were checked against their prescriptions, which were available for them to consult. Knowledge levels were attributed on the basis of the interviewees’ replies. The knowledge level attributed was ‘good’ when the patient’s reply was completely correct, ‘regular’ when it was partially correct, and ‘poor’ when incorrect or if the patient did not provide answers. Regular and poor knowledge levels were defined as insufficient knowledge. 

 Fourteen questions, with closed dichotomous responses (yes/no), were administered to determine what information about the precautions needed when on warfarin treatment had been provided to the interviewees. The questions were based on the guidance provided in Brazil’s national prescribing guidelines (*Formulário Terapêutico Nacional* ) 10 and the country’s manual for stroke care routines (*Manual de Rotinas para Atenção ao AVC*). 11 Each positive response scores one point, up to a maximum score of fourteen. The amount of information provided to patients was classified as good if the score was 10 points or more, and insufficient if not. This cutoff point of approximately 70% has also been used in other studies. 12 ^-^ 14

 Adherence was measured using the Portuguese translation of the (8-item Morisky Medication Adherence Scale, MMAS-8), which has been validated. 15 ^,^ 16 The MMAS-8 was originally developed to assess treatment adherence in patients with systemic arterial hypertension, but Wang et al. 17 and Mayet 18 have validated it for assessing treatment adherence in patients taking warfarin. To date, there are no published studies conducted in Brazil using the MMAS-8 to assess adherence to oral anticoagulation treatment. 

 The MMAS-8 comprises eight questions related to adherence behavior, seven with closed dichotomous responses (yes/no) and the last with a five-point response scale: never, almost never, sometimes, frequently, and always. Each response indicating adherence scores one point. High adherence was defined as a score of eight points, moderate adherence as a score of seven or six points, and low adherence as a score of five points or fewer. For this study, patients with high adherence were considered adherent, and the sensitivity and specificity of the MMAS-8 were calculated using INR as the gold standard, in line with Mayet. 18

 The PT tests, used to provide the INR value, were conducted by a third-party laboratory, and blood samples were collected at home. According to both Brazilian 19 and international anticoagulation guidelines, 1 ^,^ 2 ^,^ 5 the therapeutic INR range recommended for the majority of indications is from 2.0 to 3.0. However, there are exceptions for some patients with prosthetic valves or patients with frequent thromboembolic events, who may need an INR value in the range of 2.5 to 3.5. 2 ^,^ 20 ^,^ 21

 In the present study, patients were considered to be within the correct therapeutic range if their INR was between 2.0 and 3.5. This range has been used previously in a study that enrolled both patients with prosthetic valves and patients without prostheses, 20 since an INR value of up to 3.5 can be tolerated without changing the warfarin dosage, even by patients whose target anticoagulation range is 2.0 to 3.0. 21 ^-^ 23 Patients with an INR value below 2.0 were considered not to be adherent to their warfarin treatment. 

 Predictive variables analyzed were sex, age (64 years or younger; over 64 years of age), educational level (up to 5 years of study; more than 5 years of study), time taking warfarin (up to 24 months; more than 24 months), indication for warfarin treatment (prosthetic valves; thromboembolic diseases and other reasons), frequency of PT testing (interval of 3 months or less; interval exceeding 3 months), level of knowledge about prescription (good; insufficient), amount of information provided by healthcare team (good; insufficient), treatment adherence (adherent; not adherent). 

 Analyses involved presentation of absolute and relative values for the study variables, expressing continuous variables as means with standard deviations. Data were analyzed estimating prevalence, prevalence ratios (PR), and 95% confidence intervals (95%CI). The chi-square test was used to test for associations between independent variables and INR values, with continuous variables dichotomized. Analyses were conducted in SPSS version 18.0, considering a significance level of 0.05. 

 The study was approved by the institution’s Research Ethics Committee and interviewees participated voluntarily, were told they could opt out at any point, and signed free and informed consent forms. The study complies with the ethical principles enshrined in National Health Council Resolution 466/2012. 

## RESULTS

 A total of 96 patients with warfarin prescriptions were identified, five of whom refused to participate in the study, thirteen could not be located, seven were no longer taking warfarin, and three had died. The study was started with 68 participants, eight of whom did not complete it. 

 The study analyzed data on 60 people, with a mean age of 65.3±13.7 years, 31 of whom were women (51.7%). Mean educational level was 5.8±4.4 years of study, mean time on warfarin was 5.8±5.0 years, and the principal indications for warfarin treatment were thromboembolic diseases, in 25 participants (41.7%); prosthetic heart valves, in 23 (38.3%); and other reasons (arrhythmia, acute myocardial infarction, or ischemic stroke), in 10 (16.7%). Two people (3.3%) did not know why they had been prescribed warfarin. 

Table 1 lists sociodemographic characteristics and the frequency of INR values beyond the therapeutic range for each category of variables. The data shown in Table 1 demonstrate that, among the variables analyzed, there was a higher prevalence of INR values outside the therapeutic range, but there were no statistically significant associations between variables and the INR value. The chi-square test also revealed no associations between the variables and the INR value (p > 0.05). 

**Table 1:** Sociodemographic characteristics and INR values outside of the therapeutic range among warfarin users treated by primary health care in the municipal district of Ijuí, RS, Brazil.

**Characteristics**	**Participants (n = 60)**	**Participants outside therapeutic range** **(n = 38)**		
	**n (%)**	**n (%)**	**Prevalence (%)**	**PR (95%CI)**
Sex				
Female	31 (51.7)	23 (60.5)	74.2	1.43 (0.95-2.15)
Male	29 (48.3)	15 (39.5)	51.7	
Age				
Up to 64 years	27 (45.0)	16 (42.1)	59.3	
Over 64 years	33 (55.0)	22 (57.9)	66.7	1.30 (0.76-1.67)
Educational level				
Up to 5 years of study	39 (65.0)	25 (65.8)	64.1	1.03 (0.69-1.56)
More than 5 years of study	21 (35.0)	13 (34.2)	61.9	
Time taking warfarin				
Up to 24 months	19 (31.7)	13 (34.2)	68.4	1.12 (0.78-1.66)
More than 24 months	41 (68.3)	25 (65.8)	60.9	
Indication for warfarin treatment				
Prosthetic valves	23 (38.3)	16 (42.1)	69.6	1.17 (0.80-1.71)
Thromboembolic and other diseases	37 (61.7)	22 (57.9)	59.4	
Frequency of PT testing				
Maximum interval of 3 months	33 (55.0)	20 (52.6)	60.6	1.10 (0.75-1.61)
Interval exceeding 3 months	27 (45.0)	18 (47.4)	66.7	
Level of knowledge about prescription				
Good	30 (50.0)	19 (50.0)	63.3	1.00 (0.68-1.47)
Insufficient	30 (50.0)	19 (50.0)	63.3	
Level of information provided by the healthcare team				
Good	10 (16.7)	5 (13.1)	50.0	
Insufficient	50 (83.3)	33 (86.8)	66.0	1.32 (0.69-2.53)
Adherence				
Adherent	8 (13.3)	6 (15.8)	75.0	0.82 (0.54-1.29)
Not adherent	52 (86.7)	32 (84.2)	61.5	

n (%): number and percentage of participants; PR (95%CI): prevalence ratio with 95% confidence interval.

 With regard to level of knowledge about prescriptions, 30 participants (50%) demonstrated a good level of knowledge and the other 30 were classified as having an insufficient level of knowledge, among whom 16 (26.7%) exhibited a regular level of knowledge and 14 (23.3%) had a poor level of knowledge. With regard to the amount of information provided to the patients by the healthcare team, 50 (83.3%) received insufficient information and just 10 (16.7%) were provided with sufficient information. Table 2 lists the items of information and the frequency with which each was provided to the patients. 

**Table 2:** Level of information provided to warfarin users by healthcare team in the municipal district of Ijuí, RS, Brazil (n = 60).

**Question: Did the doctor or the healthcare team that treat you tell you any of the following items of information about treatment with warfarin?**	**Participants informed** **n (%)**
1. Not to take medicines via intramuscular injection during treatment.	9 (15.0)
2. Do not take other medicines on your own initiative, particularly anti-inflammatories.	30 (50.0)
3. In case of spontaneous bleeding (gums, urinary, genital, etc.), inform your doctor and seek medical care immediately.	28 (46.7)
4. Always tell any dentist or doctor you see that you take warfarin.	28 (46.7)
5. Use closed, non-slip footwear to avoid falls and injuries.	18 (30.0)
6. Use a rubber mat in the bathroom to avoid falls.	16 (26.7)
7. Take a prothrombin time test (INR) at least every 3 months.	30 (50.0)
8. Seek medical care immediately if you have an intense headache or stomach pains.	11 (18.3)
9. Control your intake of foods rich in vitamin K (broccoli, cabbage, collard greens, spinach, and certain vegetable oils) and multivitamins and nutritional supplements containing vitamin K.	15 (25.0)
10. Avoid sports or other activities that can cause injury.	16 (26.7)
11. Take care when brushing teeth and shaving and to avoid injuries to the head and body.	16 (26.7)
12. Do not take other medicines without consulting your doctor or pharmacists, because warfarin has a high probability of adverse reactions and interacts with many medicines.	18 (30.0)
13. What is the specific recommendation about the INR value you should have?	26 (43.3)
14. What dietary precautions should you take?	20 (33.3)

n (%): number and percentage of participants.

 When asked if they had been given information about the INR value, 26 participants (43.3%) replied that they had, but only 19 (31.7%) were able to correctly state what their ideal therapeutic range was. When asked about the frequency with which they took a PT test to control INR, 33 participants (55.0%) reported that they were tested at least once every 3 months, 16 (26.7%) stated that they took the test at intervals exceeding 3 months, and another 11 (18.3%) stated that they were not tested. 

 However, when requested to show their most recent PT test result, to confirm the INR values, only 15 participants (25.0%) had an up-to-date test result, 22 (36.7%) provided the result of a test conducted more than 3 months previously, 11 (18.3%) patients who said they underwent testing could not provide the result, and another 12 (20.0%) said they had not been tested. In order to confirm INR values, it was decided to conduct PT tests for all participants, with samples collected at home. 

 The results of these PT tests showed that just 22 participants (36.7%) were within the therapeutic range, with INR values of 2.0 to 3.5. Nine (15.0%) of the 38 participants (63.3%) who were outside of the therapeutic range had INR values exceeding 3.5 and the remaining 29 (48.3%) had INR values below 2.0, and were considered not to be adherent to warfarin treatment. 

 Administration of the MMAS-8 showed that just eight participants (13.3%) exhibited high adherence. Thirty-seven participants (61.7%) with adherence moderate and 15 (25.0%) with low adherence were considered non-adherent. Mean MMAS-8 score was 6.1±1.7, on a scale from zero to eight points. Taking the INR value as gold standard, MMAS-8 had good sensitivity, since it defined 84.2% of the participants who were outside of the therapeutic range as non-adherent to treatment. Its positive predictive value showed that 61.5% of the non-adherent participants were outside of the therapeutic range. However, the MMAS-8 exhibited low specificity, since just 9.1% of the participants who were within the therapeutic range were defined as adherent to treatment according to the MMAS-8. The negative predictive value demonstrated that 25.0% of participants defined as adherent according to the MMAS-8 were within the therapeutic range. Table 3 lists the MMAS-8 questions used to evaluate adherence to treatment with warfarin and the number of participants whose replies were indicative of adherence. 

**Table 3:** Questions asked to check for treatment adherence and number of participants with replies indicative of adherence to warfarin treatment (n = 60).

**Questions asked about treatment with warfarin**	**Reply indicating adherence**	**n (%)**
1. Do you sometimes forget to take your medicine?	No	32 (53.3)
2. Over the past 2 weeks, were there any days when you did not take your medicine?	No	53 (88.3)
3. Have you ever stopped taking your medicine or reduced the dose without telling your doctor because you felt worse when you took it?	No	44 (73.3)
4. When you travel or leave home, do you sometimes forget to take your medicine with you?	No	44 (73.3)
5. Did you take your medicine yesterday?	Yes	53 (88.3)
6. When you feel like your disease is under control, do you sometimes stop taking your medicine?	No	53 (88.3)
7. Have you ever felt inconvenienced by having to correctly follow your treatment?	No	39 (65.0)
8. How often do you have difficulty remembering to take your medicine?	Never or almost never	46 (76.7)

n (%): number and percentage of participants.

## DISCUSSION

 The results of this study indicate a lack of information provided by the healthcare team to patients on warfarin. A mean of 4.7±3.8 of the 14 instructions recommended by Brazil’s national prescribing guidelines (*Formulário Terapêutico Nacional* ) 10 and the country’s manual for stroke care routines (*Manual de Rotinas para Atenção ao AVC*) 11 were actually provided to the patients. Just 10 participants (16.7%) stated they had been given at least 70% of these instructions and were defined as having been provided with a good level of information. Other studies, conducted in anticoagulation clinic settings, have classified from 13.3% to 74.1% 6 ^,^ 7 ^,^ 12 ^-^ 14 ^,^ 24 of participants as having a good level of knowledge about oral anticoagulation treatment. Studies by Henn et al. 6 and Rocha et al. 7 had similar data to the present study for variables such as sex, age, educational level, and time taking warfarin, but they investigated samples of 120 and 110 patients respectively, in which 64.1% and 36.4% were classified as having a good level of knowledge, considering a higher cutoff point (80%) for this classification. Variations between studies could be related to methodological differences and the fact that the present study was conducted in PHC, i.e., not at specialist anticoagulation clinics. 

 The results indicate that all of the recommended information was provided with low frequency by the healthcare team, since a maximum of 50% of participants stated they had received each item of information. Half of the participants did not even know that they should not take medications on their own initiative, not even anti-inflammatories, which are one of the classes most used in self-medication and can increase the anticoagulant effects of warfarin. The lack of this information, which is essential to treatment safety, exposes warfarin users to adverse events. Also important is patients’ lack of information regarding their medical prescriptions, since even though they were given the opportunity to consult their prescriptions when they were being interviewed, only 50% were able to state the correct warfarin usage as prescribed. 

 The low levels of information observed in this study may indicate failures in the health system with respect to the care provided to patients and to communication of information. Structural and procedural problems may compromise the availability of more rational and humanized care at PHC services 25 and the quality of the information provided to patients by the healthcare team. 

 Studies undertaken in specialist anticoagulation clinics using the MMAS-8 exhibited a higher percentage of patients with treatment adherence (from 34.5% 17 to 46.4% 18 ). Other studies, also conducted in specialist services, but using the 4-item Morisky Medication Adherence Scale (MMAS-4), 26 found treatment adherence rates of 39% to 50%. 3 ^,^ 7 ^,^ 27

 The MMAS-8 is a low-cost method that is easy to administer and offers good sensitivity but low specificity, i.e., a low proportion of individuals within the therapeutic range were defined as adherent to treatment. Of the 22 participants who were within the correct therapeutic range, just two were defined as adherent according to the MMAS-8 (9.1%). One of the reasons could be the high cutoff point of the MMAS-8, since by only defining as adherent those participants who demonstrate adherent behavior for all eight questions on the scale, it reduces the percentage of individuals who can be classified as adherent. Thus, in the present study, the MMAS-8 did not prove to be a good method for detecting adherence to treatment with warfarin. 

 It was observed that 67.3% of the interviewees were outside of the target therapeutic range. Studies conducted in oral anticoagulation settings have identified similar frequencies, from 64 to 75%, 3 ^,^ 6 ^,^ 8 ^,^ 18 contradicting the expectation that care at a specialist clinic would result in better control of anticoagulation levels, with lower frequencies of patients outside the therapeutic range. 

 The low frequency of INR values within the therapeutic range suggests that there are difficulties with maintaining adequate anticoagulation levels, whether or not patients are seen at a specialist clinic. This difficulty could be because of several factors that could influence the INR value, such as taking the pills at irregular times, inadequate dosage adjustment, variations between different manufacturers, individual factors related with genetics, diet, body mass, hepatic function, and drug metabolism. 6 ^,^ 21

 In the present study, 50% of the participants stated they had been informed that they should test PT every 3 months, but only 25.0% had an up-to-date PT result and 68.3% did not even know what their target therapeutic range was. It is possible that the low frequency of patients who know this information is related to the frequency with which the PT test is performed and the frequency of medical consultations. Specialist clinics conduct periodic monitoring and so their patients are more likely to know about PT testing and the therapeutic target range, as can be observed from the study by Rocha et al., in which 62.7% of patients treated at an anticoagulation clinic were able to state their therapeutic target. 7

 There were no associations between INR values and the variables analyzed. This result is in line with other studies, that also failed to detect significant associations, not even with variables related to knowledge 3 ^,^ 6 ^,^ 14 or treatment adherence. 3 ^,^ 18

 One limitations affecting this study is related to conducting PT tests once only. The number of participants was low; but attempts were made to enroll all patients cared for by PHC who were taking warfarin in the municipal district of Ijuí. Memory bias and use of self-report information may have influenced the results for the level of information provided to patients and their treatment adherence. It is possible that during administration of the questionnaire some of the patients did not remember items of information that had been provided by the healthcare team. 

## CONCLUSIONS

 Warfarin is considered a potentially dangerous medication, in both hospital and clinical settings, although its efficacy and safety are established. Information on how to use this medication and on the importance of adherence to treatment are therefore essential. 25 Similarly, instructions on the precautions that must be taken with warfarin treatment are especially necessary to ensure patient safety and prevent complications. 

 It was observed that there is a need to improve the quality of information provided by the healthcare team to patients treated with warfarin, to encourage treatment adherence, and to improve anticoagulation monitoring, especially for patients treated in PHC, i.e. who do not attend specialist clinics, in order to achieve better healthcare provision, which should be integral, multidisciplinary, humanized, and regular, to ensure patient safety. 
