# Supplementary material for: Knowledge and information levels and adherence to oral anticoagulant therapy with warfarin in patients attending primary health care services
Source: J Vasc Bras. 2018 Apr-Jun;17(2):109–16. doi: 10.1590/1677-5449.012017 (PMC6205714; doi:10.1590/1677-5449.012017)
Supplement: Translation

# Translation: Nível de informação e adesão à terapia de anticoagulação oral com varfarina em pacientes acompanhados em ambulatório de atenção primária à saúde

**Authors:** Thais Furtado de Souza, Christiane Fátima Colet, Isabela Heineck

**Affiliations:** 1 Universidade Federal do Rio Grande do Sul – UFRGS, Programa de Pós-graduação em Assistência Farmacêutica, Porto Alegre, RS, Brasil.; 2 Universidade Federal do Rio Grande do Sul – UFRGS, Programa de Pós-graduação em Ciências Farmacêuticas, Porto Alegre, RS, Brasil.

**Keywords:** varfarina, atenção primária à saúde, informação, adesão à medicação, coeficiente internacional normatizado

## Abstract

**Contexto:**

A anticoagulação oral com varfarina é usada por milhões de pacientes em todo o mundo, apresentando segurança e eficácia bem estabelecidas. Ainda assim, na atenção primária à saúde, os anticoagulantes estão entre as classes de medicamentos mais associadas a erros de medicação fatais.

**Objetivo:**

Verificar o nível de informação e a adesão ao tratamento com varfarina em pacientes acompanhados em ambulatório de atenção primária à saúde.

**Método:**

Foi realizado um estudo transversal de uma coorte prospectiva com 60 pacientes em uso de varfarina no município de Ijuí, Rio Grande do Sul. Utilizou-se questionário para verificar o nível de informações dos usuários quanto à prescrição e o nível das informações prestadas pela equipe de saúde aos usuários. A Escala de Adesão Terapêutica de Morisky de Oito Itens (MMAS-8) e o coeficiente internacional normatizado (*international normalized ratio*, INR) foram usados para verificar a adesão ao tratamento.

**Resultados:**

Os resultados foram expressos em valores absolutos e relativos e razão de prevalência, com seu respectivo intervalo de confiança de 95%. Verificou-se que 83,3% dos participantes tiveram nível de informação insuficiente prestada pela equipe de saúde, 50,0% não souberam informar sobre o uso correto do medicamento, 86,7% foram não aderentes ao tratamento segundo a MMAS-8, e 63,3% estavam fora do intervalo terapêutico adequado.

**Conclusão:**

Neste estudo, observou-se a necessidade de melhorar a qualidade das informações prestadas aos usuários e criar estratégias para adesão ao tratamento, visando à segurança do paciente em tratamento com varfarina na atenção primária à saúde.

## INTRODUÇÃO

 A anticoagulação oral com varfarina é usada por milhões de pessoas em todo o mundo e tem segurança e eficácia bem estabelecidas 1 ^,^ 2 . No entanto, seu uso exige acompanhamento clínico e laboratorial rigoroso, com avaliação da equipe multidisciplinar 3 . Os pacientes devem monitorar regularmente os níveis de coagulação sanguínea, por meio do exame do tempo de protrombina (TP), expresso pelo coeficiente internacional normatizado (*International Normalized Ratio*, INR) 4 , o qual deve permanecer dentro do intervalo terapêutico estabelecido para reduzir o risco de complicações tromboembólicas ou hemorrágicas 4 . 

 Na atenção primária à saúde (APS), os anticoagulantes orais (ACOs), em especial a varfarina, estão entre as classes de medicamentos mais associadas a erros de medicação fatais, muitas vezes resultantes de monitorização laboratorial inadequada, interações medicamentosas significativas, falhas no conhecimento técnico dos profissionais envolvidos e orientação insuficiente aos pacientes 5 . 

 O sucesso e a segurança dos ACOs dependem da educação do paciente, da boa adesão ao tratamento e da comunicação entre o paciente e a equipe responsável pelo seu atendimento clínico 2 ^,^ 5 . No entanto, as publicações sobre ACOs enfatizam principalmente eventos adversos, como hemorragia e eventos tromboembólicos, sem mencionar a qualidade do atendimento 5 . No Brasil, os estudos que tratam sobre o nível de informação ou sobre a adesão à terapia de anticoagulação oral estão centrados em pacientes acompanhados em ambulatórios especializados 3 ^,^ 4 ^,^ 6 ^-^ 8 . 

 Nesse contexto, o presente estudo foi realizado com pacientes em terapia de anticoagulação oral com varfarina, sem acompanhamento em ambulatório especializado, atendidos na APS do município de Ijuí (RS), com o objetivo de verificar o nível de informações dos usuários quanto à prescrição de varfarina e o nível das informações prestadas pela equipe de saúde a esses usuários sobre cuidados no tratamento, além de determinar a adesão ao tratamento com varfarina. 

## MÉTODOS

 Foi realizado um estudo transversal de uma coorte prospectiva, com dados coletados entre abril e julho de 2014. Os pacientes da coorte foram acompanhados mensalmente, pelo período de 18 meses, de abril de 2014 a outubro de 2015. Procurou-se incluir todos os pacientes que retiraram o medicamento varfarina nas unidades de saúde do município de Ijuí. O município tem população de 79.396 habitantes 9 e a APS municipal conta com 15 unidades de atendimento, sendo sete Unidades Básicas de Saúde e oito Unidades de Estratégia de Saúde da Família, nas quais se realiza a dispensação de medicamentos. 

 Os pacientes em uso de varfarina foram identificados a partir de um levantamento das receitas atendidas nas unidades de saúde do município. Para isso, realizou-se a busca e a análise das prescrições arquivadas na Farmácia Central do município para identificar prescrições de varfarina. 

 Para coleta de dados, foi utilizado um questionário, o qual abordou características sociodemográficas e questões relacionadas ao tratamento com varfarina. O nível de informação quanto à prescrição médica foi verificado a partir de uma pergunta aberta, na qual os entrevistados explicaram como deveriam usar o medicamento. As respostas dos entrevistados foram confrontadas com a respectiva prescrição, a qual poderia ser usada para consulta. Foram atribuídos níveis de informação conforme as respostas dos entrevistados. O nível de informação foi considerado bom quando houve acerto total, regular quando houve acerto parcial, e ruim em caso de erro ou não resposta. Os níveis de informação regular e ruim foram considerados como nível insuficiente. 

 Quatorze questões, com respostas fechadas de caráter dicotômico (sim/não), foram aplicadas para verificar quais informações os entrevistados haviam recebido sobre os cuidados necessários durante o tratamento com varfarina. As questões foram formuladas com base nas orientações do Formulário Terapêutico Nacional 10 e do Manual de Rotinas para Atenção ao AVC 11 . Cada resposta positiva contou um ponto, e o escore máximo foi de quatorze pontos. O nível das informações prestadas aos pacientes foi classificado como bom em caso de pontuação igual ou superior a 10 pontos, e como insuficiente em caso de pontuações inferiores. Esse ponto de corte, de aproximadamente 70%, também foi utilizado em outros estudos 12 ^-^ 14 . 

 Para determinar a adesão foi utilizada a Escala de Adesão Terapêutica de Morisky de Oito Itens (*8-item Morisky Medication Adherence Scale*, MMAS-8), que está validada em português 15 ^,^ 16 . Originalmente, a MMAS-8 foi elaborada para verificar a adesão ao tratamento em pacientes portadores de hipertensão arterial sistêmica, porém Wang et al. 17 e Mayet 18 a validaram para verificar a adesão ao tratamento em paciente que utilizavam varfarina. Até o momento, não há publicação no Brasil utilizando a MMAS-8 para verificar a adesão à terapia de anticoagulação oral. 

 A MMAS-8 utiliza oito perguntas relacionadas ao comportamento aderente, com sete perguntas com respostas fechadas de caráter dicotômico (sim/não) e a última respondida segundo uma escala de cinco opções: nunca, quase nunca, às vezes, frequentemente e sempre. Para cada resposta que demonstrasse a adesão, foi atribuído um ponto. Foi considerado alta adesão um escore de oito pontos, adesão moderada um escore de sete ou seis pontos, e baixa adesão um escore de cinco pontos ou menos. O presente estudo considerou como aderentes aqueles pacientes com alta adesão, e calculou a sensibilidade e a especificidade da MMAS-8 utilizando o valor de INR como padrão, seguindo o estudo de Mayet 18 . 

 O exame do TP, expresso pelo valor de INR, foi feito em laboratório contratado, sendo a coleta realizada em domicílio. Segundo as diretrizes para anticoagulação, em nível nacional 19 e internacional 1 ^,^ 2 ^,^ 5 , o intervalo terapêutico recomendado de INR está entre 2,0 e 3,0 para a maioria das indicações. Porém, exceções ocorrem para alguns pacientes portadores de prótese valvar ou pacientes com eventos tromboembólicos frequentes, que podem necessitar um valor de INR entre 2,5 e 3,5 2 ^,^ 20 ^,^ 21 . 

 No presente estudo, foram considerados dentro do intervalo terapêutico adequado pacientes cujo valor de INR ficou entre 2,0 e 3,5. Esse mesmo intervalo foi usado em um estudo que incluiu tanto pacientes com prótese valvar como pacientes sem prótese 20 , uma vez que o valor INR até 3,5 pode ser tolerado sem alteração da dose de varfarina, mesmo que a meta de anticoagulação do paciente seja o intervalo entre 2,0 e 3,0 21 ^-^ 23 . Os pacientes cujo valor de INR foi inferior a 2,0 foram considerados não aderentes ao tratamento com varfarina. 

 As variáveis preditoras foram sexo, idade (até 64 anos, acima de 64 anos), escolaridade (até 5 anos de estudo, acima de 5 anos de estudo), tempo de uso de varfarina (até 24 meses, acima de 24 meses), indicação para o uso de varfarina (prótese valvar, doenças tromboembólicas e outros motivos), frequência de realização do TP (intervalo máximo de 3 meses, intervalo superior a 3 meses), nível de informação quanto à prescrição (bom, insuficiente), nível das informações prestadas pela equipe de saúde (bom, insuficiente), adesão ao tratamento (aderente, não aderente). 

 As análises envolveram a apresentação de valores absolutos e relativos das variáveis em estudo, sendo utilizados a média e o desvio padrão para as variáveis contínuas. Os dados foram analisados estimando-se a prevalência, a razão de prevalência (RP) e o intervalo de confiança de 95% (IC95%). O teste qui-quadrado foi usado para verificar a associação entre as variáveis independentes e o valor de INR, sendo as variáveis contínuas dicotomizadas. As análises foram realizadas no programa SPSS versão 18.0, considerando um nível de significância de 0,05. 

 O estudo foi aprovado pelo Comitê de Ética em Pesquisa da instituição, a participação dos entrevistados foi voluntária, podendo ser interrompida a qualquer momento, e foi assinado o Termo de Consentimento Livre e Esclarecido. A pesquisa seguiu preconizados princípios éticos preconizados pela Resolução 466/2012 do Conselho Nacional de Saúde. 

## RESULTADOS

 Foram encontrados 96 usuários com prescrição de varfarina, dos quais cinco recusaram-se a participar do estudo, treze não foram localizados, sete não estavam mais utilizando varfarina e três haviam falecido. O estudo foi iniciado com 68 participantes, dos quais oito não o concluíram. 

 Foram estudadas 60 pessoas, com idade média de 65,3±13,7 anos, sendo 31 mulheres (51,7%). O nível de escolaridade foi de 5,8±4,4 anos de estudo, o tempo médio em uso de varfarina foi de 5,8±5,0 anos, e as principais indicações para o uso de varfarina foram doenças tromboembólicas, em 25 participantes (41,7%); uso de prótese valvar cardíaca, em 23 (38,3%); e outros motivos (arritmia, infarto agudo do miocárdio ou acidente vascular cerebral isquêmico), em 10 (16,7%). Além disso, duas pessoas (3,3%) não souberam informar a indicação de uso. 

 A Tabela 1 apresenta as características sociodemográficas e a frequência com que o valor de INR ficou fora do intervalo terapêutico estabelecido em cada categoria das variáveis. Os dados apresentados na Tabela 1 demonstram que, entre as variáveis analisadas, houve maior prevalência para o valor de INR fora da faixa terapêutica, porém não houve associação estatisticamente significativa entre as variáveis e o valor de INR. O teste qui-quadrado também não demonstrou associação entre as variáveis e o valor de INR (p > 0,05). 

**Tabela 1:** Características sociodemográficas e valor de INR fora da faixa terapêutica entre usuários de varfarina na atenção primária à saúde no município de Ijuí (RS).

**Características**	**Participantes (n = 60)**	**Participantes fora do intervalo terapêutico** **(n = 38)**		
	**n (%)**	**n (%)**	**Prevalência (%)**	**RP (IC95%)**
Sexo				
Feminino	31 (51,7)	23 (60,5)	74,2	1,43 (0,95-2,15)
Masculino	29 (48,3)	15 (39,5)	51,7	
Idade				
Até 64 anos	27 (45,0)	16 (42,1)	59,3	
Acima de 64 anos	33 (55,0)	22 (57,9)	66,7	1,30 (0,76-1,67)
Escolaridade				
Até 5 anos de estudo	39 (65,0)	25 (65,8)	64,1	1,03 (0,69-1,56)
Acima de 5 anos de estudo	21 (35,0)	13 (34,2)	61,9	
Tempo em uso de varfarina				
Até 24 meses	19 (31,7)	13 (34,2)	68,4	1,12 (0,78-1,66)
Acima de 24 meses	41 (68,3)	25 (65,8)	60,9	
Indicação do uso de varfarina				
Prótese valvar	23 (38,3)	16 (42,1)	69,6	1,17 (0,80-1,71)
Doenças tromboembólicas e outros	37 (61,7)	22 (57,9)	59,4	
Frequência do exame de TP				
Intervalo máximo de 3 meses	33 (55,0)	20 (52,6)	60,6	1,10 (0,75-1,61)
Intervalo superior a 3 meses	27 (45,0)	18 (47,4)	66,7	
Nível de informação quanto à prescrição				
Bom	30 (50,0)	19 (50,0)	63,3	1,00 (0,68-1,47)
Insuficiente	30 (50,0)	19 (50,0)	63,3	
Nível das informações prestadas pela equipe de saúde				
Bom	10 (16,7)	5 (13,1)	50,0	
Insuficiente	50 (83,3)	33 (86,8)	66,0	1,32 (0,69-2,53)
Adesão				
Aderente	8 (13,3)	6 (15,8)	75,0	0,82 (0,54-1,29)
Não aderente	52 (86,7)	32 (84,2)	61,5	

n (%): número e percentual de participantes; RP (IC95%): razão de prevalência com intervalo de confiança de 95%.

 Quanto ao nível de informação sobre a prescrição, 30 participantes (50%) demonstraram bom nível de informação e os outros 30 foram classificados com nível de informação insuficiente, sendo que 16 (26,7%) apresentaram nível de informação regular e 14 (23,3%) apresentarem nível de informação ruim. Quanto ao nível das informações prestadas aos pacientes pela equipe de saúde, 50 (83,3%) tiveram nível de informação insuficiente e apenas 10 (16,7%) atingiram nível de informação suficiente. A Tabela 2 apresenta as informações e a frequência com que as informações teriam sido prestadas aos pacientes. 

**Tabela 2:** Nível das informações prestada aos usuários de varfarina pela equipe de saúde no município de Ijuí (RS) (n = 60).

**Questão: O médico ou a equipe de saúde que o atende lhe prestou alguma destas informações quanto ao tratamento com varfarina?**	**Participantes informados** **n (%)**	
1. Não usar medicamento injetável via intramuscular durante o tratamento.		9 (15,0)
2. Não usar medicamentos por conta própria, principalmente anti-inflamatórios.		30 (50,0)
3. Em caso de sangramentos espontâneos (gengival, urinário, genital, etc.), comunicar o médico e procurar atendimento imediato.		28 (46,7)
4. Comunicar sempre o uso de varfarina ao dentista ou médico que lhe atender.		28 (46,7)
5. Usar sapato fechado e não escorregadio para evitar quedas e machucados.		18 (30,0)
6. Usar tapete de borracha no banheiro para evitar quedas.		16 (26,7)
7. Fazer o exame do tempo de protrombina (INR) com intervalos máximos de 3 meses.		30 (50,0)
8. Em caso de dor intensa de cabeça ou dor de barriga, procurar atendimento imediato.		11 (18,3)
9. Ingerir com moderação alimentos ricos em vitamina K (brócolis, repolho, couve, espinafre ou certos óleos vegetais), além de complexos vitamínicos e suplementos nutricionais com vitamina K.		15 (25,0)
10. Evitar a prática de esportes ou atividades que possam causar lesões.		16 (26,7)
11. Cuidados com a escovação dental, com o ato de barbear, e para evitar lesões na cabeça e no corpo.		16 (26,7)
12. Não usar outros medicamentos sem consultar médico ou farmacêutico, pois varfarina tem alta probabilidade de reação adversa e interage com diversos medicamentos.		18 (30,0)
13. Qual a indicação específica sobre o valor de INR que você deve ter.		26 (43,3)
14. Cuidados quanto à alimentação que você deveria ter.		20 (33,3)

n (%): número e percentual de participantes.

 Questionados se haviam recebido informação sobre o valor de INR, 26 participantes (43,3%) responderam afirmativamente, mas apenas 19 (31,7%) souberam informar corretamente qual o intervalo terapêutico esperado. Quando questionados sobre a frequência com que realizavam o exame de TP para controle do valor de INR, 33 participantes (55,0%) relataram fazer o exame pelo menos uma vez a cada 3 meses, 16 (26,7%) afirmaram fazer o exame com um intervalo superior a 3 meses, e outros 11 (18,3%) afirmaram que não realizavam o exame. 

 No entanto, quando foi solicitada a apresentação dos últimos resultados de TP, para verificação do valor de INR, apenas 15 participantes (25,0%) apresentaram o resultado atualizado do exame, 22 (36,7%) apresentaram resultado de exame realizado há mais de 3 meses, 11 (18,3%) que afirmaram realizar o exame não apresentaram o resultado, e outros 12 (20,0%) disseram não realizar o exame. Dessa forma, para verificar o valor de INR, optou-se por realizar o exame de TP em todos os participantes, mediante coleta domiciliar. 

 Realizado o TP, verificou-se que apenas 22 participantes (36,7%) estavam dentro do intervalo terapêutico, com valor de INR entre 2,0 e 3,5. Entre os 38 participantes (63,3%) que estavam fora do intervalo terapêutico esperado, nove (15,0%) tiveram valor de INR superior a 3,5, e os outros 29 (48,3%) tiveram valor de INR inferior a 2,0, sendo considerados não aderentes ao tratamento com varfarina. 

 Aplicada a MMAS-8, verificou-se que apenas oito participantes (13,3%) apresentaram alta adesão. Foram considerados não aderentes 37 participantes (61,7%) com adesão moderada e 15 (25,0%) com baixa adesão A pontuação média da MMAS-8 foi de 6,1±1,7 pontos em uma escala de zero a oito pontos. Utilizando o valor de INR como padrão, a MMAS-8 apresentou boa sensibilidade, pois 84,2% dos participantes que estavam fora do intervalo terapêutico não foram aderentes ao tratamento. O valor preditivo positivo demonstrou que 61,5% dos participantes não aderentes estavam fora do intervalo terapêutico. Entretanto, a MMAS-8 apresentou baixa especificidade, pois apenas 9,1% dos participantes que estavam dentro do intervalo terapêutico demonstraram-se aderentes ao tratamento de acordo com a MMAS-8. O valor preditivo negativo demonstrou que 25,0% dos participantes aderentes de acordo com a MMAS-8 estavam dentro do intervalo terapêutico. A Tabela 3 apresenta as questões da MMAS-8 para avaliar a adesão ao tratamento com varfarina e o número de participantes com respostas indicativas de adesão. 

**Tabela 3:** Questões realizadas para verificar a adesão ao tratamento e número de participantes com respostas indicativas de adesão ao tratamento com varfarina (n = 60).

**Questões realizadas quanto ao tratamento com varfarina**	**Resposta para adesão**	**n (%)**
1. Você às vezes se esquece de tomar o medicamento?	Não	32 (53,3)
2. Nas 2 últimas semanas, houve algum dia em que você não tomou ao medicamento?	Não	53 (88,3)
3. Você já parou de tomar o medicamento ou diminuiu a dose sem avisar seu médico porque se sentia pior quando os tomava?	Não	44 (73,3)
4. Quando você viaja ou sai de casa, às vezes esquece de levar o medicamento?	Não	44 (73,3)
5. Você tomou o medicamento ontem?	Sim	53 (88,3)
6. Quando sente que sua doença está controlada, você às vezes para de tomar o medicamento?	Não	53 (88,3)
7. Você já se sentiu incomodado por seguir corretamente o seu tratamento?	Não	39 (65,0)
8. Com que frequência você tem dificuldades para se lembrar de tomar o medicamento?	Nunca ou quase nunca	46 (76,7)

n (%): número e percentual de participantes.

## DISCUSSÃO

 Os resultados do presente estudo indicam uma carência de informações prestadas pela equipe de saúde aos pacientes em uso de varfarina. Dentre as 14 orientações recomendadas pelo Formulário Terapêutico Nacional 10 e pelo Manual de Rotinas para Atenção ao AVC 11 , uma média de 4,7±3,8 teriam sido prestadas aos pacientes. Apenas 10 participantes (16,7%) afirmaram ter recebido pelo menos 70% das informações, os quais foram considerados com bom nível de informação. Outros estudos, realizados em ambulatório de anticoagulação, tiveram entre 13,3% e 74,1% 6 ^,^ 7 ^,^ 12 ^-^ 14 ^,^ 24 dos participantes classificados com bom nível de informação quanto à terapia de anticoagulação oral. Os estudos de Henn et al. 6 e de Rocha et al. 7 tiveram características como sexo, idade, escolaridade e tempo de uso de varfarina semelhantes às do presente estudo, porém avaliaram amostras de 120 e 110 pacientes respectivamente, em que 64,1% e 36,4% foram classificados com bom nível de informação, considerando um ponto de corte maior (80%) para essa classificação. As variações entre os estudos podem estar relacionadas a diferenças metodológicas e ao fato de o presente estudo ter sido realizado na APS, ou seja, fora de um serviço especializado em anticoagulação. 

 Os resultados indicam que todas as informações recomendadas teriam sido prestadas com baixa frequência pela equipe de saúde, pois o máximo de 50% dos participantes afirmou ter recebido cada uma das informações. Metade dos participantes sequer sabia que não deveria usar medicamentos por conta própria, nem mesmo anti-inflamatórios, uma das classes mais usadas na automedicação e que pode aumentar o efeito anticoagulante de varfarina. O desconhecimento dessas informações, essenciais para a segurança do tratamento, expõe os usuários a eventos adversos. Pode-se considerar também uma carência de informação quanto à prescrição médica, pois, mesmo com a possibilidade de consultar a prescrição no momento da pesquisa, apenas 50% souberam informar o uso correto de varfarina conforme o prescrito. 

 Os baixos níveis de informação encontrados neste estudo podem indicar falhas no sistema de saúde no que diz respeito à atenção prestada ao usuário e à comunicação das informações. Problemas estruturais e de funcionamento podem prejudicar a disponibilidade de um atendimento mais racional e humanizado nos serviços de APS 25 , bem como a qualidade das informações prestadas pela equipe de saúde aos usuários. 

 Estudos realizados em ambulatório especializado em anticoagulação e que utilizaram a MMAS-8 apresentaram um percentual maior de pacientes com adesão ao tratamento (entre 34,5% 17 e 46,4% 18 ). Outros estudos, também realizados em serviço especializado, porém utilizando a Escala de Adesão Terapêutica de Morisky de Quatro Itens ( *4-item Morisky Medication Adherence Scale* MMAS-4) 26 , encontraram taxas de adesão ao tratamento entre 39% e 50% 3 ^,^ 7 ^,^ 27 . 

 A MMAS-8 é um método de baixo custo, de fácil aplicação e apresentou boa sensibilidade mas baixa especificidade, ou seja, baixa proporção de indivíduos dentro da faixa terapêutica aderentes ao tratamento. Dentre os 22 participantes que estavam dentro da faixa terapêutica adequada, apenas dois demonstraram adesão de acordo com a MMAS-8 (9,1%). Uma das razões pode ser o alto ponto de corte da MMAS-8, pois, ao considerar aderentes apenas aqueles que demonstrem comportamento aderente para as oito questões da escala, reduz o percentual de indivíduos que poderiam ser classificados como aderentes. Dessa forma, no presente estudo a MMAS-8 não demonstrou ser um bom método para verificar a adesão ao tratamento com varfarina. 

 Observou-se que 67,3% dos entrevistados estavam fora do intervalo terapêutico alvo. Outros estudos realizados em ambulatório de anticoagulação oral identificaram frequências semelhantes, entre 64 e 75% 3 ^,^ 6 ^,^ 8 ^,^ 18 , contrariando a expectativa de que o acompanhamento em ambulatório especializado poderia resultar em um melhor controle dos níveis de anticoagulação, com menor frequência de pacientes fora do intervalo terapêutico. 

 A baixa frequência de valores de INR dentro do intervalo terapêutico sugere a dificuldade de manter níveis adequados de anticoagulação, sendo os pacientes acompanhados em ambulatório especializado ou não. Essa dificuldade pode ocorrer devido aos diversos fatores que podem influenciar no valor de INR, tais como horário irregular de tomada dos comprimidos, ajustes inadequados de doses, variações entre os fabricantes, fatores individuais relacionados com a genética, dieta, massa corporal, função hepática e metabolismo do fármaco 6 ^,^ 21 . 

 No presente estudo, 50% dos participantes afirmaram ter sido informados que deveriam fazer o TP a cada 3 meses, mas apenas 25,0% apresentaram um resultado atualizado do TP e 68,3% não souberam nem mesmo informar o seu intervalo terapêutico alvo. É possível que a baixa frequência dessa informação esteja relacionada com a frequência de realização do exame de TP e de consultas médicas. Em ambulatórios especializados, há acompanhamento periódico; assim, os pacientes têm mais chances de tomar conhecimento a respeito do exame de TP e do alvo terapêutico, conforme pode ser observado no estudo de Rocha et al., em que 62,7% dos pacientes acompanhados em ambulatório de anticoagulação souberam informar o seu alvo terapêutico 7 . 

 Não houve associação entre o valor de INR e as variáveis analisadas Esse resultado está de acordo com outros estudos, que também não demonstraram associação significativa nem mesmo com as variáveis relacionadas ao conhecimento 3 ^,^ 6 ^,^ 14 ou à adesão ao tratamento 3 ^,^ 18 . 

 As limitações deste estudo estiveram relacionadas à realização do TP uma única vez. O número de participantes foi baixo; no entanto, procurou-se incluir todos os pacientes atendidos na APS que estariam em uso de varfarina no município de Ijuí. Viés de memória e o uso de informações autorreferidas podem ter influenciado os resultados do nível de informação prestada aos usuários e da adesão ao tratamento. É possível que alguns usuários não tenham recordado, durante a aplicação do questionário, informações que tivessem sido prestadas pela equipe de saúde. 

## CONCLUSÃO

 A varfarina é considerada um medicamento potencialmente perigoso, em âmbito hospitalar e ambulatorial, embora tenha eficácia e segurança estabelecidas. Dessa forma, informações sobre como utilizar o medicamento e sobre a importância de cumprir o tratamento são imprescindíveis 25 . Da mesma forma, orientações sobre cuidados são especialmente necessárias no tratamento com varfarina, visando à segurança do paciente e à prevenção de complicações. 

 Verifica-se a necessidade de melhoria na qualidade das informações prestadas pela equipe de saúde aos pacientes em tratamento com varfarina, bem como de incentivo à adesão ao tratamento e melhor monitoramento da anticoagulação, especialmente naqueles acompanhados na APS, ou seja, fora de ambulatórios especializados, visando ao atendimento qualificado, que deve ser prestado de forma integral, multidisciplinar, humanizada e com frequência regular, para segurança do paciente.
